# The Unspecific Side of Acquired Immunity Against Infectious Disease: Causes and Consequences

**DOI:** 10.3389/fmicb.2015.01525

**Published:** 2016-01-11

**Authors:** Eric Muraille

**Affiliations:** Laboratoire de Parasitologie, Faculté de Médecine, Université Libre de BruxellesBruxelles, Belgium

**Keywords:** infectious diseases, microbiota, acquired immunity, cross-protection, heterologous immunity, mackaness effect, trained immunity, nonspecific effects of vaccines

## Abstract

Acquired immunity against infectious disease (AIID) has long been considered as strictly dependent on the B and T lymphocytes of the adaptive immune system. Consequently, AIID has been viewed as highly specific to the antigens expressed by pathogens. However, a growing body of data motivates revision of this central paradigm of immunology. Unrelated past infection, vaccination, and chronic infection have been found to induce cross-protection against numerous pathogens. These observations can be partially explained by the poly-specificity of antigenic T and B receptors, the Mackaness effect and trained immunity. In addition, numerous studies highlight the importance of microbiota composition on resistance to infectious disease via direct competition or modulation of the immune response. All of these data support the idea that a non-negligible part of AIID in nature can be nonspecific to the pathogens encountered and even of the antigens expressed by pathogens. As this protection may be dependent on the private T and B repertoires produced by the random rearrangement of genes, past immune history, chronic infection, and microbiota composition, it is largely unpredictable at the individual level. However, we can reasonably expect that a better understanding of the underlying mechanisms will allow us to statistically predict cross-protection at the population level. From an evolutionary perspective, selection of immune mechanisms allowing for partially nonspecific AIID would appear to be advantageous against highly polymorphic and rapidly evolving pathogens. This new emerging paradigm may have several important consequences on our understanding of individual infectious disease susceptibility and our conception of tolerance, vaccination and therapeutic strategies against infection and cancer. It also underscores the importance of viewing the microbiota and persisting infectious agents as integral parts of the immune system.

## Acquired immunity against infectious disease

Long ago, physicians observed that people who had recovered from the plague never got it again and displayed “acquired immunity” against infectious disease (AIID). Early vaccination techniques developed by Jenner were based on these observations and immunology stemmed, in large part, from the necessity to understand how vaccines work in order to improve their efficacy and their safety. For decades, AIID has been explained to immunology students as strictly dependent on the adaptive immune system composed of B and T lymphocytes expressing highly specific antigenic receptors. As a result, the development of vaccines has been closely linked to the identification of immunodominant antigens expressed by pathogens, with vaccination being presented as an infectious disease-specific intervention. However, a growing body of data from epidemiology, vaccinology, and experimental immunology research supports the idea that AIID can also be frequently nonspecific to the antigens expressed by pathogens. Depending on the case, this phenomenon has been explained by (i) cross-protection induced by vaccination or past infection, (ii) the “Mackaness effect” resulting from unrelated chronic infection, and (iii) microbiota-mediated protection.

### Nonspecific protective effects of vaccination

Numerous epidemiological and experimental studies have reported that previous exposure to unrelated infectious agents can greatly alter the host's immune response to an infection and induce a state of protective immunity, frequently termed “heterologous immunity” (reviewed in Welsh and Selin, 2002; Selin et al., 2011; Benn et al., 2013; Aaby et al., 2014). For example, epidemiological studies have shown that individuals vaccinated with vaccinia virus were less susceptible to infectious diseases such as measles, scarlet fever, whooping cough, and syphilis when compared to non-vaccinated persons (Mayr, 2004). In experimental studies, BCG vaccination protected not only against mycobacteria, but also against secondary infections with *Listeria monocytogenes* (Blanden et al., 1969), *Salmonella typhimurium* (Blanden et al., 1969), *Staphylococcus aureus* (Sher et al., 1975), *Candida albicans* (Sher et al., 1975; Van't Wout et al., 1992), *Plasmodium yoelii* (Matsumoto et al., 2000; Parra et al., 2013), and *Schistosoma mansoni* (Tribouley et al., 1978). Of course, nonspecific vaccine effects are not always positive. For example, while the diphtheria-tetanus-pertussis (DTP) vaccine protects against the three targeted diseases, it has been documented to increase female mortality from other infectious diseases (Aaby et al., 2012). The mechanisms underlying this negative effect are still unknown and remain to be investigated. On the whole, these data suggest that, in addition to their antigen- or target-specific effects, live vaccines “*have nonspecific effects that may be just as important, or even more important*” (Benn et al., 2013). As expected, similar experimental observations have been made with non-attenuated, fully virulent pathogens. For example, memory cytotoxic CD8^+^ T lymphocytes induced by lymphocytic choriomeningitis virus (LCMV) infection are reactivated by the Pichinde virus and vaccinia virus, demonstrating that prior immunity to a specific virus could modulate future primary immune responses to a second, unrelated virus (Selin et al., 1994). We should also note that a comparison of homologous and heterologous protection showed that heterologous protection was generally considerably less than the almost total protection seen after challenge with a homologous infectious agent (Frenkel and Caldwell, 1975).

### Chronic infection and the “mackaness effect”

**Figure 1:**
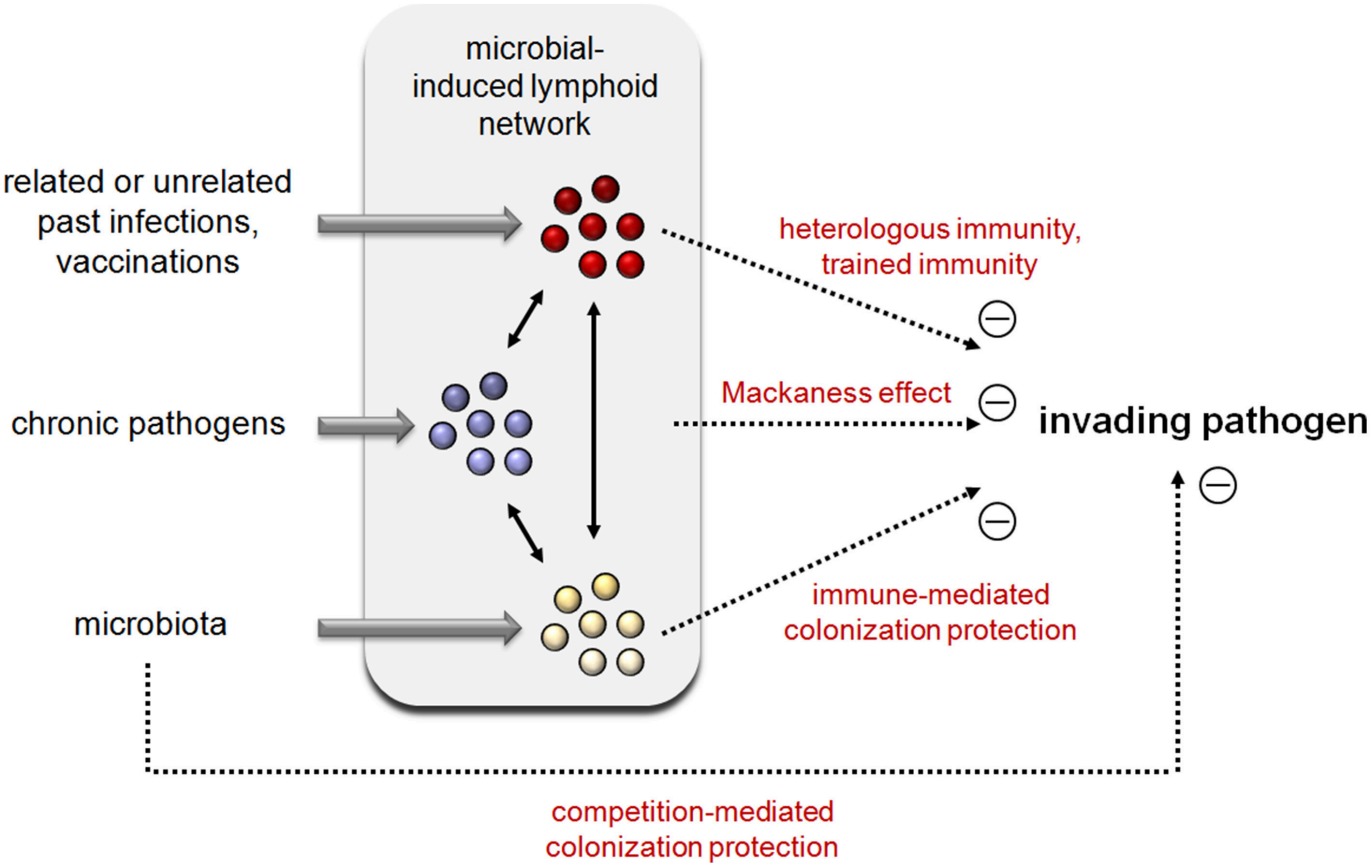
**Integrated view of the impact of past infection, chronic pathogen infection, and the microbiota on new invading pathogens**.

Research has long neglected the study of “slow infection” (associated with a long incubation period) and chronic infection by stealth or silent pathogens, such as *herpesviruses, cytomegalovirus, Brucella*, and *Bartonella*, which are the causative agents of several under-diagnosed chronic diseases constituting the most common infectious diseases. However, it has now become clear that persistence of a pathogen, even at very low levels, can affect the ability of the immune system to react to a new unrelated infection (reviewed in Stelekati and Wherry, 2012). Chronic infection can reduce or enhance the ability to control unrelated pathogens, a phenomenon termed the “Mackaness effect” in reference to the seminal work of Mackaness (Mackaness, 1964; Blanden et al., 1969) in 1964 demonstrating cross-protection between *L. monocytogenes, Brucella abortus* and *Mycobacterium tuberculosis* in mice (see Figure 1). More recent studies showed that herpes virus infection can confer beneficial protection against *L. monocytogenes* and *Yersinia pestis* (Barton et al., 2007), *Helicobacter pylori* infection is associated with protection against tuberculosis (Perry et al., 2010) and cytomegalovirus infection enhances the immune response to influenza vaccination (Furman et al., 2015). The precise molecular mechanisms involved in the Mackaness effect have yet to be elucidated. However, some aspects of the innate immune response, such as polarization of macrophages (Potian et al., 2011), stable histone changes in innate immune effector cells (epigenetic reprogramming) (Kleinnijenhuis et al., 2012) and nutritional immunity (Nairz et al., 2010; Cassat and Skaar, 2013), may account for the protective effect of chronic infection.

### Microbiota-mediated protection against infection

Microbes colonize mammalian hosts immediately after birth, forming a resident-specific microbial community including bacteria, fungi, and viruses. This is referred to as the “microbiota.” Numerous authors (Kitano and Oda, 2006; Cerf-Bensussan and Gaboriau-Routhiau, 2010) have proposed that the microbiota can be considered as an integral part of the immune system. In support of this view, it is well known that germ-free mice are highly susceptible to infection (reviewed in Cerf-Bensussan and Gaboriau-Routhiau, 2010) and that the microbiota is required for adequate immune system maturation (Ivanov et al., 2009; Buffie and Pamer, 2013) and can contribute to the control of infectious microorganisms (Ivanov et al., 2009; Buffie and Pamer, 2013; Kamada et al., 2013). How the microbiota prevents pathogen colonization has been studied for many years, and the mechanisms involved largely fall into two categories: direct interactions between the microbiota and pathogens (competition-mediated colonization protection) and microbiota-mediated enhancement of host defense mechanisms (immune-mediated colonization protection). The microbiota can compete with pathogens to acquire nutrients, produce toxic compounds such as H_2_O_2_ or target structures that mediate adherence to the epithelium of the competing microorganism (reviewed in Bosch et al., 2013). The microbiota can also affect the responsiveness of the innate immune system (Wang et al., 2013), induce a cross-reacting immune repertoire able to recognize pathogens (reviewed in Buffie and Pamer, 2013) and favor the persistence of peripheral memory T cells (Tanaka et al., 2007), probably via cross-reactivity between the antigens recognized by memory T cells and the variety of antigens derived from the members of the microbiota. Systematic studies of the dynamic interactions between the microbiota and pathogens at the host mucosal surface could help develop predictive models of cross-protection (Bosch et al., 2013).

## The nonspecific side of the adaptive immune response

All of the data discussed above indicate that the efficiency of the individual immune response to fight infectious microorganisms is largely dependent on endogenous (mutualistic and parasitic) microorganisms and the past immune history of the host (Figure 1). Recent discoveries regarding the poly-specificity of antigenic receptors, the important role of innate immune cells in regulation of the adaptive response and trained immunity have provided a better understanding of the underlying mechanisms of cross-protection.

### The “poly-specificity” of B and T lymphocytes

The “clonal selection hypothesis” developed by Jerne in 1954, which is based on the “Lock and Key” model of Emil Fischer, proposes that each lymphocyte bears a single type of antigen receptor with unique specificity (the “one clonotype–one specificity” paradigm). Under that theory, stimulation of this receptor is required for activation of the cell and entrance of an antigen into the body results in the selection of only a few B lymphocytes that recognize the antigen and produce a corresponding antibody to destroy it. However, since this pioneering time, the “poly-specificity” (also termed poly-reactivity, plasticity or degeneracy) of B cell receptor (BCR) and T cell receptor (TCR) [functionally the ability of a single receptor to specifically recognize many different antigens, as discussed in (Wucherpfennig et al., 2007)] has been well documented [reviewed in (Notkins, 2004) for BCRs and in (Wucherpfennig, 2004; Eisen and Chakraborty, 2010; Sewell, 2012) for TCRs]. Poly-specificity of the BCR has been mostly observed with natural antibodies, such as IgM in particular, that dominate the newborn B cell repertoire (Chen et al., 1998). Poly-specific BCRs bind to self-antigens as well as to a variety of bacteria and viruses (Oldstone, 2014), suggesting that they may be involved in preventing the dissemination of pathogens. Their biological importance has been demonstrated in antibody-deficient mice, which can be protected against lethal infections via the transfer of serum from antibody-competent naive animals (Ochsenbein et al., 1999; Jayasekera et al., 2007; Zola et al., 2009). Concerning the TCR, it has been estimated, for example, that a single patient-derived autoimmune CD8 T cell clone recognizes over one million distinct decamer peptides in the context of a single MHC class I molecule (Wooldridge et al., 2012). As discussed by the authors, the poly-specificity of TCRs appears to be crucial for the interaction of an individual's limited TCR repertoire (<10^8^ T cell clones) with the >10^15^ peptide MHC constituting the vast array of different potential ligands. Experiments have shown that peptides do not necessarily need to show high sequence homology to cross-react with the same T cell (Joshi et al., 2001). Consequently, even the injection of a specific (purified and well defined) antigen can induce the activation and expansion of a lymphocyte population able to potentially interact with more than just the antigen itself. As the reactivity of this population is not easy to predict, it is no longer appropriate to use the term “specific” (in the meaning of “definite” or “precise”) to describe the immune response induced by a specific antigen. In addition, the physiological state of memory T cells is such that they are primed for activation, and they have been observed to be productively stimulated by a peptide concentration that is 50 times lower than the one required for the stimulation of naive T cells (Curtsinger et al., 1998). So, it would not be surprising if a memory T cell could be stimulated by a cross-reactive peptide with substantially lower affinity for the TCR than the “original” peptide that created the memory T-cell pool. A theoretical study suggests that although cross-reactivity is a rare event for immunologically naive individuals, the probability of cross-reactive memory T cells becomes very high following successive infections (Zarnitsyna et al., 2013). B and T lymphocytes also express a great number of pattern recognition receptors (PRRs), such as Toll-like receptors (TLRs), that are able to interact with microbial associated structures or stress-induced molecules and induce or modulate their activation (Rawlings et al., 2012). Polyclonal stimulation by a microbial product such as CpG DNA may participate in the long-term maintenance of memory B cells (Bernasconi et al., 2002). Bystander infections can also promote optimal memory T cell responses by providing inflammatory signals such as IL-15 that are critical for the rapid proliferation of memory CD8^+^ T cells (Richer et al., 2015). A recent study showed that inflammatory monocyte-derived IL-15 and IL-18 can activate memory CD8^+^ T cells in a bystander manner in the apparent absence of TCR ligation (Soudja et al., 2012). In summary, an accumulating mass of evidence demonstrates that lymphocyte activation via antigen receptors may also not be “exquisitely antigen-specific” (Alberts et al., 2002) as previously considered and could furthermore be dependent on nonspecific innate immune receptors.

### Orchestration of the immune response

The metaphor by which lymphocytes act as a conductor orchestrating the immune response has been used widely. This idea progressively changed at the end of the twentieth century when immunologists fully realized the crucial role of nonspecific innate immunity in regulating lymphocyte activity and the immune response. In fact, a growing body of data suggests that stromal cells, innate immune cells and “non-conventional” lymphocytes, such as γδT cells, natural killer T (NKT) cells, and B1 lymphocytes, play an important role in regulation of both the primary and memory immune response to infectious agents (reviewed in Degli-Esposti and Smyth, 2005; Mueller and Germain, 2009; Bonneville et al., 2010). These non-conventional lymphocytes usually display low specificity and frequently appear to be pre-selected to recognize pathogen-associated molecular patterns (PAMPs). γδT cells recognize a plethora of factors induced by stress conditions, few of which are characterized (Bonneville et al., 2010). NKT cells are activated by lipoprotein PAMPs presented by CD1 receptors (Rossjohn et al., 2012). B1 lymphocytes are frequently poly-specifics and seem to be selected to recognize both self-antigens and PAMPs (Baumgarth, 2011).

### Trained immunity

Until less than a decade ago, there was a general assumption that the B and T lymphocytes of the adaptive immune system were the only components able to generate memory cells, and mount recall memory responses (Murphy et al., 2008). Several recent studies have challenged this dogma (reviewed in Netea et al., 2011; Min-Oo et al., 2013; Martin, 2014), suggesting that the innate immune system displays adaptive properties. Natural Killer (NK) cells (that do not express specific antigen receptors) can autonomously retain a memory of past antigen encounters and mediate more robust secondary responses (Cooper et al., 2009). In some cases, protection has been associated with the clonal expansion of NK cells (Keppel et al., 2013). Until now, it has not been clear whether the functional antigen specificity of NK memory is comparable to that of T and B lymphocyte-mediated memory. Several studies also provide evidence that monocytes exposed *in vivo* to pathogens (Quintin et al., 2012) mount protective recall responses to re-infection, suggesting that even cells derived from the myeloid lineage in mammals may possess features of adaptive immunity. This phenomenon seems to be linked with epigenetic modifications, as stable histone methylation changes have been observed in the genome of innate memory cells (Quintin et al., 2012). The term “*trained immunity*” has been proposed (Netea et al., 2011) for the persistent enhanced state of the innate immune response following exposure to certain infectious agents, which may result in increased resistance to related or unrelated pathogens. As expected, a part of the cross-protection induced by vaccines seems to be dependent on trained immunity (reviewed in Blok et al., 2015). Interestingly, although only recently described, this phenomenon seems to be very common among living organisms. Plants, which possess only an innate immune system, also display an enhanced state of resistance and faster and stronger responses following infection (Fu and Dong, 2013). The underlying mechanisms seem also to include epigenetic modifications.

The mechanisms explaining cross-protective immunity are not limited to poly-specificity of lymphocytes, the Mackaness reaction, trained immunity, or microbiota-mediated protection. Infection can durably remodel mucosal tissues, thus favoring or impairing immune responses against unrelated pathogens (reviewed in Didierlaurent et al., 2007; Foo and Phipps, 2010). For example, in the lung, successive infections modify epithelium adherence and change the lymphatic network and the frequency of inducible bronchus-associated lymphoid tissue (iBALT). In addition, it would be wise to consider each infection as a unique phenomenon, as each pathogen displays particular immune escape mechanisms directly affecting the immune system. For example, a recent study (Mina et al., 2015) has demonstrated that a large part of nonspecific measles vaccine benefits come from the ability of the vaccine to impair measles-induced immune suppression characterized by systemic depletion of lymphocytes and reduced innate immune cell proliferation. By preventing measles-associated immune memory loss, measles vaccination also induces polymicrobial herd immunity.

## A new complex ecological paradigm of immunity emerges

### The immune system: a nonlinear, interactive, and evolving network

Viewed as a whole, previous data support the idea that a non-negligible part of AIID is nonspecific to the antigens expressed by pathogens and is dependent on the past and present interactions of the host immune system with its environment. The ecological vision of the host protective immune response as dependent on multiple environmental factors is not new. In 1966, Fazekas de St Groth et al. already wrote that “*Response to vaccine depends not only on the nature of the antigen itself but also on the immunological history of the recipient*” and introduced the phenomenon of “original antigenic sin” to describe the inability of an experimented immune system to produce antibodies in response to a new antigen (Fazekas de St Groth Webster, 1966). In the same way, the “hygiene hypothesis” was proposed by Strachan (1989) to explain the rapid rise of asthma during the twentieth century. The original formulation of the hygiene hypothesis states that a lower incidence of infection in early childhood could affect the maturation of immune responses and favor the development of allergic diseases. During the last decade, this hypothesis has been progressively extended (Oikonomopoulou et al., 2013) to the development of autoimmune diseases and cancer, popularizing the idea that immune responses against pathogens, allergens, or tumors can influence each other nonlinearly. If we accept that immune cross-protection is more common in nature than previously expected, we can no longer look at vaccination or infection events independently of each other. Experimental and epidemiologic studies on cross-protection suggest that memory lymphocytes do not form fixed and isolated clusters of cells but rather an interactive and evolving cell nonlinear network displaying the properties of “adaptive complex systems” (CASs) (Holland, 2006; Brownlee, 2007) described in systems biology. CASs are open and dynamic nonlinear networks of interdependent agents, constantly acting and reacting to what the other agents are doing and to forces external to the system. As a result of these permanent complex interactions, CASs exist in a state of dynamic flux and change constantly and discontinuously. Antigenic challenge can alter the reactivity of the immune network by modifying the frequency of T and B lymphocytes and their polarization. The trained immunity phenomenon suggests that the innate immune system can also participate in this network, memorize past experiments and durably affect the polarization and reactivity of lymphocytes.

### The microbiota and pathogens form integral parts of the host immune system

Mounting evidence supports the view that eukaryotic hosts and their microbiota have co-evolved toward mutualistic interactions benefiting each partner. This vision suggests that the microbiota and the host form a superorganism (Eberl, 2010) (also termed metaorganism; Bosch and McFall-Ngai, 2011) where the immune system plays not a “military role” (fighting well defined enemies) but a “police role” (penalizing parasitic/selfish comportments) to shape homeostasis within this consortium (Muraille, 2013). As previously discussed, the microbiota plays undeniable direct and indirect roles in the control of infectious microorganisms (Ivanov et al., 2009; Buffie and Pamer, 2013; Kamada et al., 2013). However, the distinction between the microbiota and pathogens is artificial and confusing as colonization by “pathogenic” bacteria or viral species is very frequent under healthy circumstances. For example, *Streptococcus pneumoniae, H. influenzae*, and *S. aureus* are commonly found in the respiratory tract of healthy individuals (reviewed in Bosch et al., 2013) and are considered as pathobionts (i.e., potentially pathological organisms which, under normal circumstances, live like commensals). In several infectious models (Belkaid et al., 2002; Obar et al., 2006; Beura et al., 2015), persisting pathogens have been described to enhance T cell memory and assure protection against secondary infection (via the concomitant immunity phenomenon). By inducing a cross-reactive repertoire, the Mackaness reaction or trained immunity, the pathogens can frequently participate in protection of the host against unrelated infectious diseases. These observations have prompted me to propose that the concept of the microbiota as forming an integral part of the host immune system (Kitano and Oda, 2006; Cerf-Bensussan and Gaboriau-Routhiau, 2010) should logically be extended to pathogens. The host, its specific microbiota and the infecting microrganisms are interdependent and form a highly interactive network. Considering each part independently of the others is a reductionist view that has led us to neglect numerous important natural phenomena such as nonspecific AIID.

Some immunologists are still reluctant to admit the possibility of a nonspecific side of AIID. All immunological textbooks cite “specificity” as a hallmark of the adaptive immune response. In fact, this paradigm derives mainly from the use of simple proteins, such as ovalbumin, to study adaptive response. The administration of simple proteins in an inbred naive mice model induces a highly reproducible, and thus predictable, immune response. Retrospectively, this approach, through successful, appears to be questionable. First of all, this type of antigen does not naturally induce an immune response. Single proteins generally need to be associated with an adjuvant, the “immunologist's dirty little secret” as termed by Janeway (1989), to induce the inflammatory reaction required for lymphocyte activation. Under natural conditions, the reaction to a simple, non-proliferating, and non-damaging molecule is a trait of an allergic or autoimmune reaction and is generally not associated with a protective immune reaction. Second, pathogens are not simple structures and their antigen composition is very complex and fluctuates widely. This feature is frequently underestimated as immunologists generally neglect the intra-species diversity of pathogens and their “within host evolution” during chronic infection (Pybus and Rambaut, 2009; Wilson, 2012). Small RNA viruses such as HIV are a well-documented example of this. These viruses express a limited number of genes, but produce, within a very short period of time, an extraordinarily high number of variants in the host, thereby generating a dynamic heterogeneous population (termed quasi-species or cloud) with high antigen complexity (Domingo et al., 2012). Similarly, bacteria can display great diversity in the host, rapidly evolve within it (Shin et al., 2004; Hoboth et al., 2009) and follow a complex cycle (Justice et al., 2004). This structural complexity is conducive to the sharing of antigenic determinants between pathogens and the probability of cross-immune reactions. Lastly, in contrast to inbred naive mice, the variability of the adaptive immune response of individuals in nature is non negligible, even against simple proteins. This characteristic is notably due to the initial variability of the adaptive immune repertoire, as described in Oudin's seminal work on idiotypy in the rabbit in 1969 (Oudin and Michel, 1969), and to the individual's immune history which shapes the BCR and TCR repertoire. Thus, a large part of the apparent specificity of the adaptive immune response derives from the systematic use of highly reductionist experimental models selected by experimenters for their reproducibility and predictability. This conclusion should not be seen as a general challenge of the reductionist approach that is an inevitable stage in biological research, but as a reminder that our perception of reality results from the way in which we study it. We must constantly ask ourselves what we are neglecting in our models and whether or not we want to refine our understanding of reality, as this may affect our results.

From an evolutionary point of view, it would appear that the selection of an immune system displaying the potential to mediate cross-protective reactions is ineluctable to counter the selective pressure of rapidly adapting pathogens displaying complex escape immune mechanisms. Antigenic variation is one of the most common escape strategies of pathogens. The possibility of antigen-unspecific activation of immune effectors can potentially offer protection against new antigenic variants of pathogens. Numerous pathogens are also able to hide their antigens from the immune system and induce local anti-inflammatory environments. The Mackaness effect induced by unrelated infection can allow for the elimination or control of these stealth pathogens. Finally, the ability of the individual immune system to integrate and process all immune experiments results in a partially unpredictable immune response against infection. This may have also favored population survival as unpredictable individual responses promote the diversity of immune responses inside a population and thereby potential herd immunity to infection (discussed in Muraille, 2014). Thus, immune cross-reactions may not be a negligible “by-product” of the immune system response, but rather one of its fundamental traits in nature. As each individual initially produces a private T and B repertoire by random rearrangement of genes (reviewed in Muraille, 2014) and displays a particular immune history and a specific microbiota (Monteiro-da-Silva et al., 2014; Sato et al., 2015), cross-protection appears to be largely unpredictable at the individual level. However, we can reasonably expect that a better understanding of the underlying mechanisms will allow us to statistically predict cross-protection at the population level and integrate this phenomenon into future vaccination strategies. The field of nonspecific AIID is still in its infancy and needs to be systematically explored.

## Practical implications and perspectives

The paradigm of the antigen specificity of AIID has dominated the field of immunology for decades. The great majority of prophylactic and therapeutic immune treatments, such as subunit vaccines, antigen-specific immunotherapies against autoimmune diseases, tumors, and allergies, successfully exploit the specific side of acquired immunity. Acceptance of the nonspecific side of acquired immunity could have several important consequences on how we conceive self-tolerance, vaccination strategies and antitumoral immunity, and highlight the importance of considering the microbiota and chronic infectious pathogens as part of the host defense system against infection under natural conditions.

### Revisiting the theory of immune tolerance

**Figure 2:**
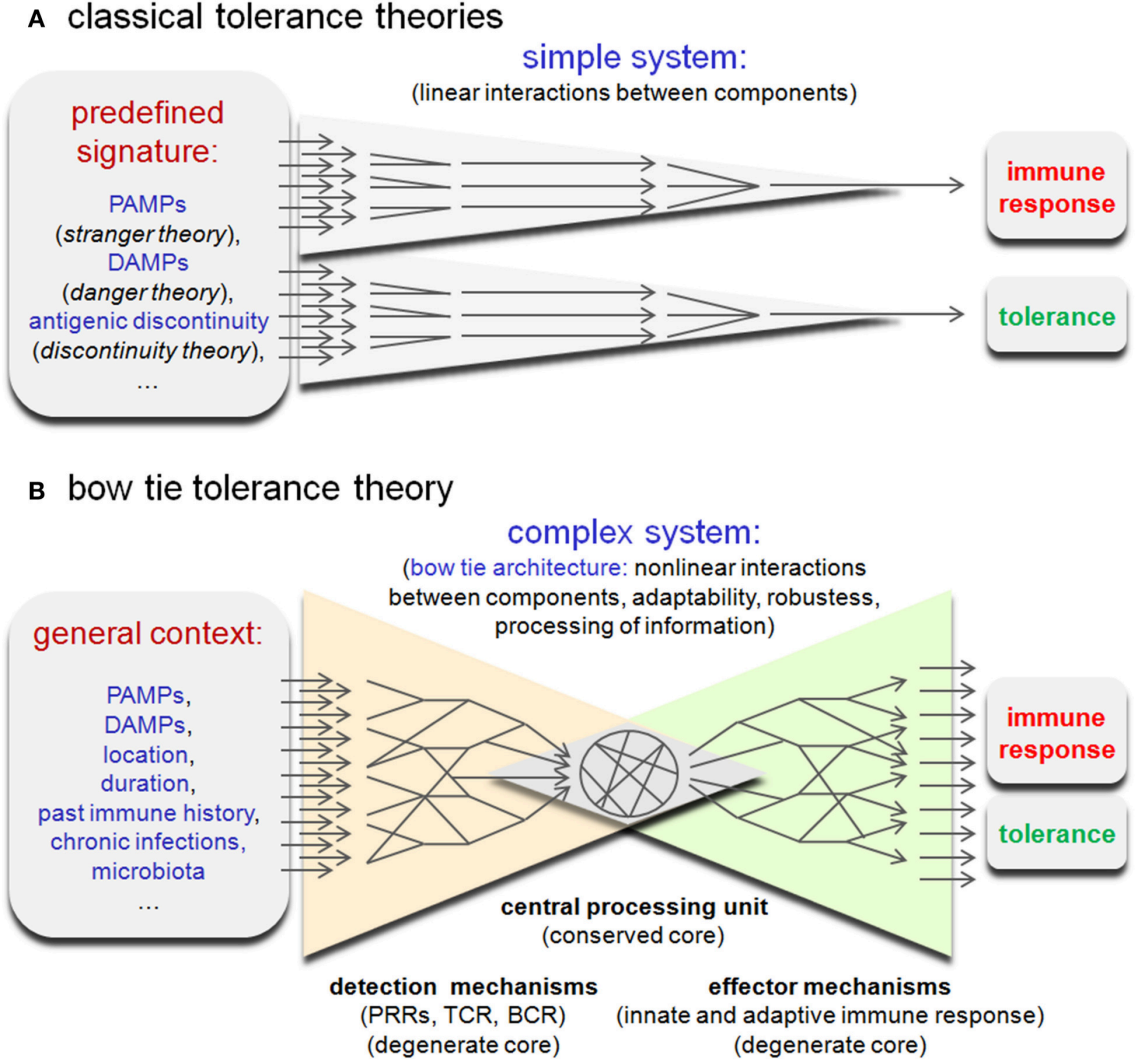
**Comparison of (A) “classical” and (B) “bow tie” tolerance theories**.

If a natural infection were to lead to an increased frequency of lymphocytes displaying a large range of reactivities, cross-reaction with a self-antigen would become frequent and inevitable. This would also mean that the pre-immune repertoire of experienced healthy individuals should display high-affinity antigen receptors specific for self-determinants. This phenomenon has been documented in healthy mice and humans (Avrameas et al., 2007; Nagele et al., 2013), but remains unexplained using the classical paradigms of tolerance. Classical self-tolerance theories propose that the choice of the immune system to tolerate or reject is based on the detection of a “simple” qualitative signal such as a microbial signature (stranger/pattern recognition theory; Janeway, 1989), damage signatures (danger theory; Matzinger, 2002), or more recently an abrupt discontinuity of the antigen signal (discontinuity theory; Pradeu et al., 2013) (Figure 2A). However, this approach does not take into consideration the connection between the components of the immune system and more particularly the highly interconnected network of the many innate and adaptive immune detectors. As discussed by Kitano (Kitano and Oda, 2006; Oda and Kitano, 2006), some parts of the immune network display a characteristic “bow tie” architecture (Figure 2B). Typically, this type of architecture detects a wide range of inputs through a degenerate detection system coupled to a “central processing unit” (conserved core), where the inputs are organized and processed. In turn, this system can provide a large variety of responses leading to non-trivial (unreliable, partially unpredictable) causal action (reviewed in Tieri et al., 2010). This suggests that immune tolerance could be the result of an elaborate computation by the immune network based on a very large set of parameters including microbial and damage signatures, but also a great number of other contextual parameters such as the location and duration of antigenic signals, the individual immune history and the general state of the host organism. In other terms, the immune network could acts as a “cognition system,” like the central nervous system, and be capable of information processing, learning, memorization, and adaptation. From this perspective, tolerance results from the interpretation of multiple signals in a general context. Of course, this does not mean that all signals have the same value. The immune system can focus on some signals, such as PAMPs or DAMPs, but the decision process remains dependant on the general context and requires information processing. This “bow tie hypothesis” fits with my proposal (Muraille, 2013) that the immune system was initially selected during evolution to allow for cooperation with the microbiota by tolerating cooperative and fighting selfish/cheater partners. It seems evident that endogenous (tumors) or exogenous (microbial pathogens) cheaters cannot be identified as such based only on a predefined signature, but on the evaluation of context-dependent behavior. This analysis requires an elaborate computation process and is indispensable to adapt to an evolving and unpredictable antigenic environment as well as to discriminate between noise and significant signals. In summary, the bow tie hypothesis suggests that a better understanding of tolerance can only come from a holistic approach to processing information networks within the immune system. In contrast, previous theories on tolerance propose mainly to focus research on the identification of specific signals (PAMPs, DAMP, etc.) supposed to regulate the tolerance process directly (linearly).

### Vaccination strategies

Historically, the great success of vaccination campaigns has been associated with the administration of live-attenuated vaccines, such as Jenner's smallpox vaccine, the Bacillus Calmette–Guérin (BCG) vaccine, Sabin's polio vaccine and Hilleman's measles, mumps, and rubella vaccine. As previously discussed, these vaccines could display important non antigen-specific protective effects dependent on the innate or adaptive immune system. Thus, some of these vaccines could have important beneficial effects on populations even if their respective target diseases have been eliminated. For example, several epidemiological studies (Aaby et al., 2006; Jensen et al., 2006; Sørup et al., 2011) showed that smallpox-vaccinated individuals are at a lower risk of hospitalization for most subgroups of infectious diseases when compared to individuals not vaccinated for smallpox. As a result, it may be important to quantify the nonspecific beneficial effects associated with each vaccine before restricting their use. Modern vaccination strategies focus on the use of “adjuvanted” subunit vaccines (Pérez et al., 2013; Reed et al., 2013; De Gregorio and Rappuoli, 2014) that drive immune responses against dominant antigens expressed with low variability by pathogens. When compared to live-attenuated vaccines, there are several strong arguments in favor of subunit vaccines: (i) their supposed safety because they exclude all risks of reversion of attenuated pathogens to a virulent form, (ii) their high specificity limiting the risk of autoimmune diseases, and (iii) the difficulty to cultivate, conserve and transport live vaccines. However, the ability of adjuvanted subunit vaccines to induce trained immunity and cross-reactive lymphocytes is poorly documented. Modern synthetic adjuvants are first of all selected for their low inflammatory potential and it is not sure that they stimulate innate immunity that is strong enough and long enough to induce trained immunity. It could be interesting to systematically investigate the ability of synthetic adjuvants to induce trained immunity. Subunit vaccine strategies generally target a reduced number of antigenic structures and limit cross reactivity, thereby completely neglecting the adaptive capacity of pathogens. Indeed, microbial pathogens frequently display a very complex and fluctuating antigen composition. In addition to presenting important intra-species diversity, they show “within host evolution” during chronic infection (Pybus and Rambaut, 2009; Wilson, 2012). Small RNA viruses such as HIV are a well-documented example of this. These viruses express a limited number of genes but produce, in a very short period of time, an extraordinarily high number of variants in the host, generating a dynamic heterogeneous population (termed quasi-species or cloud) with high antigen complexity (Domingo et al., 2012). Similarly, bacteria can display great diversity in the host and rapidly evolve within it (Shin et al., 2004; Hoboth et al., 2009). A stable specific selective pressure is unable to control rapidly evolving pathogens for long. This could explain why subunit vaccine strategies have failed for decades to control numerous pandemics such as those associated with HIV, tuberculosis and malaria. In comparison, live vaccines display wide coverage against a multiplicity of antigens and are able to better cope with pathogen strain polymorphisms and host genetic restrictions. Thus, rational replacement of old vaccines with modern versions should be based on the assessment of both the specific and nonspecific benefits of each vaccine. A better understanding and exploitation of the cross-protection conferred by vaccines could open up new avenues in vaccine research, particularly in the development of multi- or poly-reactive vaccines. Along these lines, the World Health Organization recognized the importance of the nonspecific effects of vaccines in April 2014 and recommended further research in this new direction (World Health Organization, 2014).

### Manipulation of the microbiota

Modification of the microbiota composition by probiotics (Alexandre et al., 2014) and microbiota transfer (Austin et al., 2014) can constitute a powerful and not yet fully explored strategy to prevent or fight infection with multiresistant or highly-variable pathogens that escape classical antibiotic therapies or vaccination. For example, recent work on *Plasmodium* infection revealed that the host's microbiota plays an important role in the control of malaria (Yilmaz et al., 2014). The authors demonstrated that both Plasmodium spp. and the human gut pathobiont *Escherichia coli* O86:B7 express α-gal, and that anti-α-gal antibodies are associated with protection against malaria transmission in humans, suggesting that gut probiotics could represent innovative tools for malaria prevention. Thus, a single α-gal displayed by a bacterium can affect resistance to a protozoon. This is yet another perfect demonstration that acquired resistance to infection can be nonspecific to the pathogen encountered. In the same way, therapeutic use of lytic bacteriophages (O'Flaherty et al., 2009) or predatory bacteria (Allen et al., 2014) to treat pathogenic bacterial infections is one approach that has great potential as a solution to the serious worldwide problem of drug-resistant bacteria. In addition to offering an alternative solution to antibiotics, these therapies allow for more selective elimination of pathogenic bacteria without deeply affecting the host microbiota and reducing its resistance to opportunist infection.

### Nonspecific immune control of tumors

Despite considerable progress in cancer genetics, the vast majority of metastatic solid tumors still remain incurable today. It has become clear that tumors are heterogeneous structures that, during development and growth, become “sculpted” or “edited” by immune responses and, as a result, pass through the “three E's” of elimination, equilibration and escape (reviewed in Gerlinger et al., 2014). This Darwinian evolution of tumors within hosts explains the short-lived effects of immunotherapeutic strategies based on one single or a few tumor antigens and the difficulty to identify biomarkers common to large groups of tumors. Genetically distinct subpopulations of tumor cells have also been observed to cooperate to promote tumor growth, progression, and maintenance (Gerlinger et al., 2014). Interestingly, epidemiological studies have clearly established an inverse relationship between acute infections and cancer development (Rastogi et al., 2008; Krone et al., 2014). Spontaneous tumor regression has been observed in association with a wide range of infectious organisms including those of bacterial, fungal, viral, and protozoal origin (Reilly and Christine, 1953; Oliveira et al., 2001; Rosenberg et al., 2002; Baird et al., 2013). Not all types of microorganisms are expected to have the same anticarcinogenic effect; for example, viral infections seem to be mainly procarcinogenic, in contrast to bacteria or parasitic worms that more frequently exhibit antitumorigenic effects. In contrast to acute infections, chronic infections can be viewed as resulting from a failed immune response and an increasing number have been associated with an elevated risk of cancer (Hoption Cann et al., 2006). There are scores of ways in which infectious diseases can alter the course of cancers, and this should remain an area of intense study. Once again, in contrast to the classical specific approach focusing on the identification of specific tumor antigens, a promising focus of research could be based on exploitation of the nonspecific protective effect of the pathogen-induced immune response. Indeed, several experimental studies (reviewed in Bolhassani and Zahedifard, 2012) suggest that at least some pathogens and live vaccines may be beneficial with respect to the subsequent risk of cancer.

### Conflict of interest statement

The author declares that the research was conducted in the absence of any commercial or financial relationships that could be construed as a potential conflict of interest.
